# Optical Dual Laser Based Sensor Denoising for OnlineMetal Sheet Flatness Measurement Using Hermite Interpolation

**DOI:** 10.3390/s20185441

**Published:** 2020-09-22

**Authors:** Marcos Alonso, Alberto Izaguirre, Imanol Andonegui, Manuel Graña

**Affiliations:** 1 Robotics and Automation Group, ECS Department, Mondragon University, 20500 Mondragon, Spain; aizagirre@mondragon.edu (A.I.); iandonegui@mondragon.edu (I.A.); 2 Computational Intelligence Group, Computer Science Faculty, University of the Basque Country, UPV/EHU, 00685 San Sebastian, Spain; manuel.grana@ehu.es

**Keywords:** cubic Hermite interpolation, laser triangulation, metal sheet, flatness measurement, roller leveling, smooth surface reconstruction, shape measurement system

## Abstract

Flatness sensors are required for quality control of metal sheets obtained from steel coils by roller leveling and cutting systems. This article presents an innovative system for real-time robust surface estimation of flattened metal sheets composed of two line lasers and a conventional 2D camera. Laser plane triangulation is used for surface height retrieval along virtual surface fibers. The dual laser allows instantaneous robust and quick estimation of the fiber height derivatives. Hermite cubic interpolation along the fibers allows real-time surface estimation and high frequency noise removal. Noise sources are the vibrations induced in the sheet by its movements during the process and some mechanical events, such as cutting into separate pieces. The system is validated on synthetic surfaces that simulate the most critical noise sources and on real data obtained from the installation of the sensor in an actual steel mill. In the comparison with conventional filtering methods, we achieve at least a 41% of improvement in the accuracy of the surface reconstruction.

## 1. Introduction

Laser based optical sensors have been widely used in industrial environments for various applications like online programming, part measurement and quality control, and part identification and localization.

The requirements for the surface quality of sheet metal products are continuously increasing. Flatness defects are a major problem in many industrial areas such as architectural panel works [1,2] and the automotive industry [3], just to name a few. If the incoming raw material has flatness defects, the manufacturer cannot correct it and must reject it or produce inferior quality products. Therefore, it is critical to ensure that the metal sheet products achieve high quality flatness, meeting customer requirements. Accurate, cost effective, and real-time surface measurement systems are necessary for steel sheet products’ online quality assurance.

The flatness of a metal sheet is its levelness when the sheet is free of tension. The main institution providing the definitions of flatness and how to measure it for steel sheet products is the American Society for Testing and Materials (ASTM), allowing purchasers and providers to understand each other using the same characterizations of flatness and the means of the detection of flatness anomalies. The two main standardized measures of flatness are the Steepness Index [4], and the I-Unit [5]. In a sheet whose surface exhibits sinusoidal waves of height *H* and period *L*, its Steepness Index value is defined as H/L. The surface analysis of a metal sheet makes a series of lengthwise virtual cuts of the sheet sample that result in narrow strips, which are commonly referred to as strip fibers. These fibers are treated as lines characterizing the profile of the surface. Using the length of one of these fibers as a reference, $L_{ref}$, the I-Unit value $I_{i}$ for an individual fiber *i*, is defined as:

(1) $$I_{i}=\Delta L_{i}/L_{ref}10^{5}$$

where $\Delta L_{i}$ is the difference between the length of the fiber *i* and the reference fiber $L_{ref}$, and the constant $10^{5}$ is a standard scaling factor.

Optical sensors based on laser line triangulation are becoming the most widespread solutions to acquire a dense 3D mapping of metal surfaces [6]. Using triangulation techniques, it is possible to compute the surface height from the laser line segmented on the image, where each column of the imaging sensor corresponds to a virtual fiber of the metal sheet. However, these measurements are sensitive to the noise produced by some other mechanical processes, e.g., degreasing, cleaning, polishing, shearing, and transporting roll systems, as well as the illumination effects from the ambient light and the speckles generated by the laser coherent light. Therefore, a high quality flatness measuring system strongly depends on the noise removal methods applied on the captured surface height profile.

**Figure 1:**
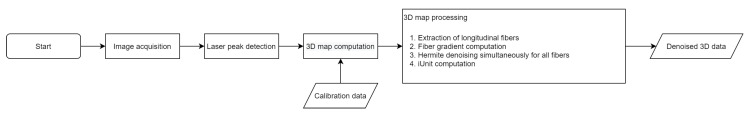
Flowchart of the computational process carried out by the sensor.

The contributions of this paper are the following: (1) the design and implementation in a real industrial operation of a surface flatness sensor that uses two parallel laser lines and an industrial high speed area scan camera providing height maps of the metal sheets moving below the sensor; and (2) a noise removal system based on Hermite polynomial interpolation that effectively removes high frequency noisy wave patterns induced in the sheet by mechanical manipulation. The flowchart in Figure 1 provides a representation of the computational process carried out by the sensor.

The filtering process is validated on synthetic and real industrial production data. This allows analyzing and filtering the information even when the surface flatness information is extremely hidden under the influence of noise sources whose amplitude exceeds several times the amplitude of the original signal. In particular, a noise source produced by a cutting station near the sensor location was removed successfully from the synthetic and real data.

The structure of the paper is as follows: Section 2 reviews the state-of-the-art techniques and devices used to measure the flatness quality of metal sheets. Section 3 focuses on the foundations of laser based optical flatness measurement devices. Section 4 gives the formal details of the numerical method used for improved noise filtering of the fiber surface height measurement maps by Hermite cubic interpolation. Section 5 discusses the sources of numerical errors. Section 6 deals with real-time surface reconstruction processing. Section 7 discusses the performance of the system using both synthetic and real data acquired on a real industrial metal sheet process. Finally, Section 8 gives some conclusions and directions for future work.

## 2. Related Works

Early metal sheet flatness measurement techniques relied on manual gauging [5], demanding skilled operators able to identify the sheet surface shape deviations for every coil and to manually operate the settings of the roll leveler machine in order to correct these deviations. The first oonline flatness measuring system, known as the stressometer, was devised using pressure transducers [7] in the late 1960s. Different kinds of shape measurement rolls, based on piezoelectric load sensors and air-bearing rotors, enabled closed loop control of the roll leveling process with reference to a target profile [8,9,10]. Whilst shape measurement rolls are currently commonly used in cold rolling mills, they are rarely used in hot, very thick rolled products, roll leveling lines, or finishing lines. The downsides of this measurement technique is that it is very sensitive to force adjustments and can cause scratching of the metal surface.

With the advent of computer vision technologies, optical flatness sensors were introduced [11] to measure manifest flatness, i.e., flatness not hidden by tension. These systems allow measuring smaller defects at higher line-speeds, enabling real-time control and a higher level of integration. The most commonly used optical surface flatness inspection systems are based on the laser triangulation principle [6]. Some designs include several laser emitters for increased robustness [12]. Alternatively, some other optical flatness measuring devices are based on ultra diffuse light or moiré pattern projection. Ultra diffuse light systems use LEDs and an elaborate system of light conductor units and mirrors, ensuring that a uniformly diffuse light bar is projected onto the material. This light bar is recorded with a matrix camera. The light intensity, the light reflection angles, and the change of both factors are evaluated while the material is moving [13]. Moiré pattern projection based systems use the output of a topographic shadow contour pattern, which must be analyzed so that the required 3D information can be formed [14]. These systems are very sensitive to motion error due to the mechanical positioning of the sheets and the shadows and light patterns created by the uncontrolled environmental lighting.

In the computer vision field, there is a long tradition of approaches dealing with surface estimation using gradient data, which are often very noisy [15,16,17,18,19]. Instances of optical based techniques proposed in the literature for the measurement of 3D geometries of objects are the use of deflectometry sensors, able to measure phase changes of light reflected from specular surfaces [20], and the reconstruction of ground surfaces from clouds of points obtained by remote sensors [21]. Integration of 1D gradient data for curve estimation is a well solved problem in the literature [22,23,24,25,26]. However, when dealing with 2D gradient data, we find at least two main kinds of procedures. When the data integration is carried out along specified lines crossing the surface in a privileged direction, we know them as local methods [22,27,28]. Their advantage is their simplicity, low computational cost, and the high resolution recovering height variations. Their disadvantage is their lack of robustness against error propagation along the line. When the data integration is carried out by variational approaches minimizing a functional with 2D support, the approach is a global method [16,29,30,31], which is more robust to error propagation due to the diverse noise sources due to the metal strip manipulation, but having much greater computational cost, which prevent real-time implementations.

We devised an optical measurement system comprised of two laser lines and a matrix camera that allows the computation of both the height and gradient information along each 1D fiber. Using a combination of both pieces of information, we developed a method capable of obtaining a precise surface reconstruction that is robust against all kinds of noise sources [32,33]. The approach based on piece-wise cubic Hermite spline interpolation is based on a global technique that allows filtering out undesirable noise sources using both surface gradient and height information. Our method addresses this problem by dividing each fiber into multiples pieces, each cubic Hermite spline minimizing both data surface and gradient information, resulting in an interpolated, smooth, globally continuous, and differentiable curve built up as a combination of cubic Hermite splines to retrieve an accurate and real-time estimation of the metal sheet surface, allowing flatness defect detection. We discarded the application of 2D interpolation methods such as bicubic interpolation splines because they do not meet the real-time requirements imposed by the industrial application that sets the stage for this article. For instance, Hermite RBF interpolations [34] are used for denoising scanner data, requiring computational times in the order of minutes, which is obviously out of question to contemplate in our application. A previously published conference paper [35] provides a rough overview discussion of our approach.

## 3. Laser Based Optical Flatness Measurement System

In this section, we address some fundamental aspects of the implemented sensor, namely the sheet-of-light triangulation principle, optical design considerations, the use of peak image intensity detector techniques, and the calibration of the camera and laser line sensor.

### 3.1. Sheet-of-Light Triangulation Principle

The sheet-of-light technique is based on the principle of triangulation [36], performing a three-dimensional reconstruction of the surface of an opaque and diffuse reflecting solid by using an area scan camera and a light line projectors. Although sheet-of-light techniques are the most commonly used for surface reconstruction, other laser projected patterns like multiple laser stripes, patterns like circles [37,38,39], concentric multiple circles, and grids may be used. The proposed flatness sensor relies on sheet-of-light triangulation technique using two laser line projectors. The camera and the line projector must be mounted so that their main axes form an angle for triangulation. The value of this triangulation angle is typically between 30${}^{\circ }$ and 60${}^{\circ }$. The projected light line defines a plane in 3D space. This plane intersects the surface of the solid under measurement, creating a profile of the surface that is visible to the camera. By moving the solid surface in front of the laser line projector, it is possible to record the whole surface of the solid. In order to increase the measurement range, a Scheimpflug configuration is used, where the detector plane has a tilted angle with respect to the imaging plane [40].

**Figure 2:**
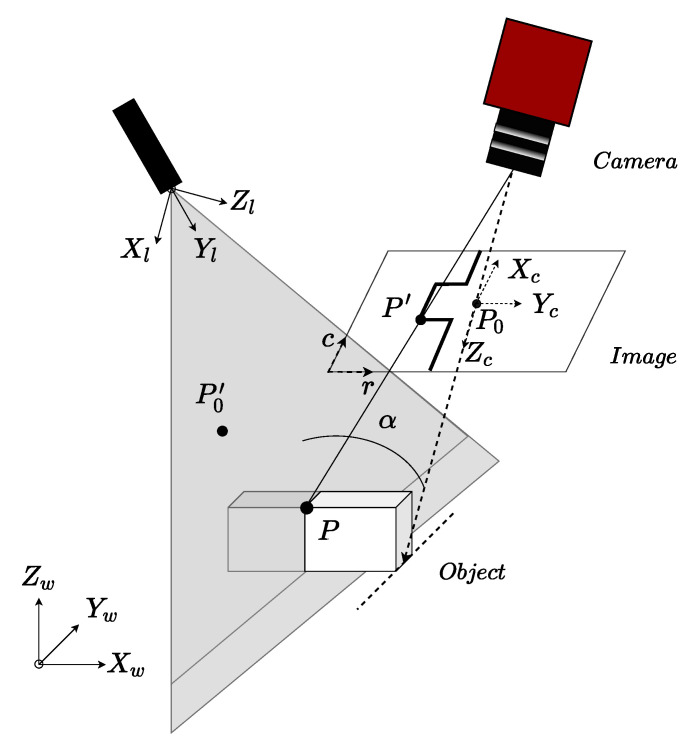
General configuration of a laser triangulation system.

The measurement principle of the sheet-of-light technique is illustrated for a single laser line triangulation setup in Figure 2, where $P=\left[X,Y,Z\right]$ is a point in the world coordinate system and $P^{{}^{\prime }}=\left[x,y\right]$ is its projection in the image plane. Equations (2) and (3) show how the 3D point P can be computed knowing its projection on the camera image plane. Equation (2) corresponds to the projected line of the observation of point P by the camera, and Equation (3) corresponds to the laser plane. Their intersection provides the desired world coordinates P.

(2) $$P=\lambda v+P_{0}$$

(3) $$n^{T}\left(P-P_{0}^{{}^{\prime }}\right)=\left[a\;b\;c\right]\left[\begin{array}{c} P_{x}\\ P_{y}\\ P_{z} \end{array}\right]+d=0$$

where $P_{0}$ is the camera optical center, v is the unitary vector between $P_{0}$ and the observed projection of the point P in the laser plane, $n={\left[a,b,c\right]}^{T}$ is the plane’s normal vector, and $P_{0}^{{}^{\prime }}$ is any point lying on the laser plane [41].

### 3.2. Speckle Noise and Spurious Reflections

Sheet-of-light laser triangulation requires robust image segmentation of the laser line in the image captured by the camera. However, spurious reflections and speckle noise make this computation difficult.

Lasers light can be focused in a narrow and bright line. Moreover, the coherent nature of laser light allows using narrow-bandwidth optical filters on the camera, removing image noise from ambient light. However, the downside of coherence is that it creates speckles in the imaging system. Speckles are interference effects of coherent light photons that travel slightly different distances from the source to the sensor, produced by surface roughness at the order of the wavelength of the incident coherent light. It gives a characteristic granular appearance when the surface is imaged under highly coherent light [42]. Speckle noise can impair the segmentation of the laser line in the image. The size of the speckle noise on the image is proportional to the laser wavelength and the lens aperture: a larger aperture generates smaller speckles in the image [43].

Spurious reflections are another big problem in the 3D surface scanning of shiny metallic surfaces. The reflected light from the targeted area of the surface may illuminate some other areas of the same surface that are detected by the segmentation of the images, causing fake measurements [44].

To minimize speckle and spurious reflections, the proposed sensor features a blue laser emitter at wavelength λ=450nm. Red laser emitters (λ=630nm) penetrate deeper into the target surfaces as compared with blue lasers. Therefore, they substantially increase the area of the blurry region corresponding to the laser line captured by the camera. Moreover, blue laser emitters generate a much more focused laser on the object surface, minimizing the light reflected back to the camera.

### 3.3. Image Intensity Peak Detector

The accuracy of a 3D reconstruction using laser linear illumination is significantly determined by the accuracy of the line segmentation in the image. Since the pattern of image intensity in the normal direction to the line has a Gaussian profile, finding the center of the line in the image corresponds to detecting the point of maximum intensity in the normal direction, also known as the laser peak, which can be detected by different algorithms using the intensity distribution along a column of the sensor image, e.g., finding the position of maximum intensity, finding thresholding points, or finding the center of gravity [45,46,47]. In the proposed sensor, a Savitzky–Golay [48] finite impulse response (FIR) differential filter is applied to the image intensity profile of the laser line, computing the zero-crossings with sub-pixel accuracy [49,50,51].

### 3.4. Sensor Calibration

#### 3.4.1. Background

The purpose of sensor calibration is to identify the camera intrinsic parameters and the mapping between the laser plane and the camera. Since the sensor consists of a laser projector and a camera, camera modeling and calibration become an integral part of the sensor calibration procedure. The conventional approach consists of using first a standard camera calibration technique [52,53] to estimate the camera parameters, then the laser plane parameters are estimated by capturing the correspondences of 3D known points and applying the least squares method [54,55,56]. The calibration methods are dependent on the form of calibration target, the method for extracting control points for camera calibration and laser plane calibration, and the calibration algorithms used, for instance the 2D plane with controlled movement [57], and the 3D target with the invariance of the cross ratio [58,59] could be used. An alternative sensor calibration approach consists of finding the mapping function between the image plane and laser plane, treating the sensor as a black box carrying out a plane mapping function. This approach requires the estimation of at least eight parameters [60] by the plane constraint methods [61] or a least squares polynomial fitting method [62].

Scanning large 3D objects, such as flattened rolled metal sheets, implies that the object is moving relative to the 3D scanner. Depth measurements computed from laser triangulation are synchronized with the motion of the metal sheet using an incremental encoder located after the roll leveler stage. This ensures a uniform data acquisition and minimizes the effect of the motion jitter.

#### 3.4.2. Proposed Calibration Method

The standard accuracy requirements in the steel processing industry are as follows: for the height, better than 2σ=0.35 mm assuming that the measurement error follows a normal distribution with zero mean and $\sigma^{2}$ variance; for the strip cross direction, better than 200 measurement zones per meter. After calibration, our sensor is capable of a height accuracy of 2σ=0.25 mm and 650 measurement zones per meter. For the proposed sensor, accuracy requirements and the cost effectiveness lead us to use a method based on direct linear mapping functions (DLM). In the pinhole camera model, the mapping between a 3D point in the world coordinate frame to the image coordinate frame is:

(4) $$sm=K\left[R,t\right]M$$

where $m={\left[\begin{array}{c} u,v,1 \end{array}\right]}^{T}$ is the vector of homogeneous coordinates in the image plane, $M={\left[\begin{array}{c} X_{w},Y{}_{w},Z_{w},1 \end{array}\right]}^{T}$ is the vector of homogeneous 3D coordinates of a point in the world coordinate frame, *s* is an arbitrary scale factor, R and t are the rotation and translation components of the transformation matrix from the world coordinate frame to the camera coordinate frame, and K is the matrix of intrinsic camera parameters:

(5) $$K=\left[\begin{array}{ccc} \alpha & r & u_{s}\\ 0 & \beta & v_{s}\\ 0 & 0 & 1 \end{array}\right]$$

then we have:

(6) $$s\left[\begin{array}{c} u\\ v\\ 1 \end{array}\right]=K\left[\begin{array}{c} r_{1},r_{2},r_{3},t \end{array}\right]\left[\begin{array}{c} X_{w}\\ Y_{w}\\ Z_{w}\\ 1 \end{array}\right]$$

assuming that all the calibration points are placed on a plane with $Y_{w}=0$ and $R=\left[r_{1},r_{2},r_{3}\right]$

(7) $$s\left[\begin{array}{c} u\\ v\\ 1 \end{array}\right]=K\left[\begin{array}{c} r_{1},r_{3},t \end{array}\right]\left[\begin{array}{c} X_{w}\\ Z_{w}\\ 1 \end{array}\right]$$

Define $H=K\left[\begin{array}{c} r_{1},r_{3},t \end{array}\right]$, $m={\left[\begin{array}{c} u,v,1 \end{array}\right]}^{T}$, and $M={\left[\begin{array}{c} X_{w},Z_{w},1 \end{array}\right]}^{T}$; we obtain the following expression for the mapping between the 2D image points and the 3D calibration planar points:

(8) sm=HM

Given a set of calibration points and their corresponding image coordinates, the transformation matrix H, also known as homography, can be estimated by solving the previous linear equation. Let us assume that the laser plane XZ is our reference coordinate system. Let $P_{li}=\begin{array}{c} \left[X_{li},Z_{li}\right] \end{array}$ be a point in the laser plane and $P_{ci}=\begin{array}{c} \left[X_{ci},Y_{ci}\right] \end{array}$ the corresponding image plane point. The mapping between these two planes is given by:

(9) $$s\left[\begin{array}{c} X_{ci}\\ Y_{ci}\\ 1 \end{array}\right]=\left[\begin{array}{ccc} h_{11} & h_{12} & h_{13}\\ h_{21} & h_{22} & h_{23}\\ h_{31} & h_{32} & h_{33} \end{array}\right]\left[\begin{array}{c} X_{w}\\ Z_{w}\\ 1 \end{array}\right]$$

We estimated the intrinsic parameters of the camera before the installation of the camera in the sensor by a previous calibration process at the lab, achieving an average retroprojection error of 0.04 pixels. We carry out the correction of the lens radial distortion so the mapping from the image plane to the laser plane becomes a linear function. The mapping transformation H can be estimated by minimal linear least squares:

(10) $$\begin{array}{ccc} sX_{ci} & = & h_{11}X_{li}+h_{12}Z_{li}+h_{13}\\ sY_{ci} & = & h_{21}X_{li}+h_{22}Z_{li}+h_{23}\\ s & = & h_{31}X_{li}+h_{32}Z_{li}+h_{33} \end{array}$$

For a set of corresponding points ${\left\{P_{li}=\begin{array}{c} \left[X_{li},Z_{li}\right] \end{array}\right\}}_{i}$ and ${\left\{P_{ci}=\begin{array}{c} \left[X_{ci},Y_{ci}\right] \end{array}\right\}}_{i}$, we can set $h_{33}=1$ without loss of generality. Rearranging the system of equations of Equation (10), we get a linear system of equations HA=0, where A and H are given by Equation (11).

(11) $$\begin{array}{ccc} A & = & {\left[\begin{array}{ccccccccc} -X_{li} & -Z_{li} & -1 & 0 & 0 & 0 & X_{ci}X_{li} & X_{ci}Z_{li} & X_{ci}\\ 0 & 0 & 0 & -X_{li} & -Z_{li} & -1 & Y_{ci}X_{li} & Y_{ci}Z_{li} & Y_{ci}\\ \vdots & \vdots & \vdots & \vdots & \vdots & \vdots & \vdots & \vdots & \vdots \\ -X_{ln} & -Z_{ln} & -1 & 0 & 0 & 0 & X_{cn}X_{ln} & X_{cn}Z_{ln} & X_{cn}\\ 0 & 0 & 0 & -X_{ln} & -Z_{ln} & -1 & Y_{ci}X_{li} & Y_{ci}Z_{li} & Y_{ci} \end{array}\right]}_{2n\times 9} \end{array}$$

(12) $$\begin{array}{ccc} H & = & {\left[\begin{array}{c} h_{11}\\ h_{12}\\ \vdots \\ h_{32}\\ h_{33} \end{array}\right]}_{1\times 2n} \end{array}$$

### 3.5. Dual Linear Laser Flatness Sensor

**Figure 3:**
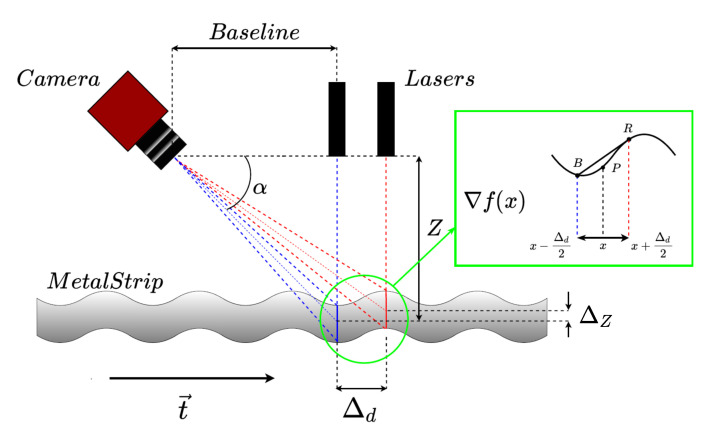
Design of the laser-camera sensor featuring two parallel laser lines allowing the computation of surface height and its gradient. The color of the representation is not related to the actual laser color, which is the same for both laser sources.

The proposed flatness sensor is comprised of two illuminating parallel linear laser sources perpendicular to the metal sheet translation axis separated by a distance $\Delta_{d}=100\;$mm from each other and a CCD camera capturing the area illuminated by the lasers, as shown in Figure 3.

This dual laser line approach allows separating high frequency vertical vibrations from low frequency flatness defects by detecting variations between lasers lines [12], as shown in the inset of Figure 3. In the implemented sensor, the baseline separation between camera and laser sources is $\Delta_{B}=900\;$mm, and the triangulation angle is $\alpha =45^{\circ }$ so that the center of the camera captures the middle of both laser lines at Z=0mm. The displacement between laser emitters is $\Delta_{d}=100\;$mm. The laser line emitters are collimated, and their wavelength is λ=450nm, while their line aperture is $90^{\circ }$. The camera features a 2048 × 2048 matrix CCD sensor, and the focal length of the lens is f=6mm, placed at Z=1140mm over a moving steel strip. The information of both laser lines allows the local gradient to be calculated as the slope of the line joining two measured points corresponding to each laser line, as seen in the inset of Figure 3. This slope remains constant no matter what vertical displacements of the metal sheet surface are produced. The height value P at point *x* is computed as P=(B+R)/2 under the assumption that the gradient does not change significantly in the interval $\left[x-\frac{\Delta d}{2},x+\frac{\Delta d}{2}\right]$.

**Figure 4:**
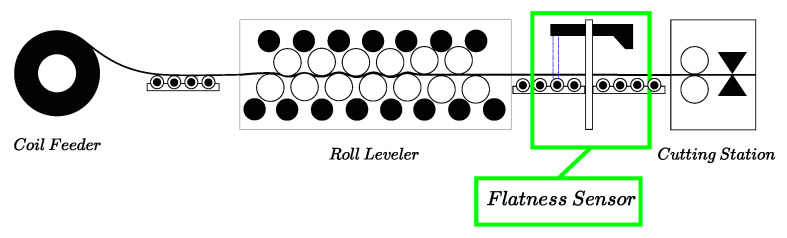
A simplified block diagram showing an industrial finishing line. The flatness sensor lies between the roll leveler and the cutting station.

**Figure 5:**
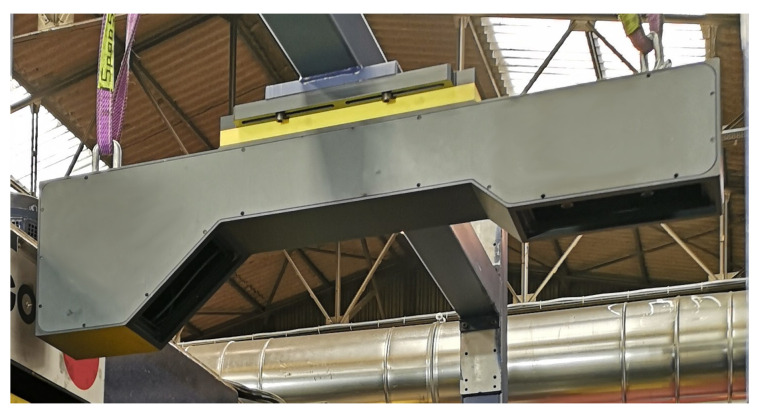
Close view of the devised sensor comprised of two linear lasers and a camera, installed over an industrial steel roll leveler processing line.

The proposed optical flatness sensor was installed in a finishing process line just after an industrial roll leveler close to a cutting station, as shown in Figure 4. The precision roller leveler has 13 rolls with a diameter of 130 mm. This leveler has the ability to apply controlled roll bend through flights of adjustable backup rollers. This line is capable of processing hot rolled materials with yield strengths in the range $\left[300,700\right]$ MPa (mild steel) and thickness ranging from 3 mm to 8 mm. The sensor shown in Figure 5 is clamped to a frame placed at the output of the leveler, and it is isolated from its structure by a polymer that efficiently reduces the vibrations produced by the manufacturing process.

## 4. Cubic Hermite Spline Interpolation with Global Continuous Derivatives

The approximation of surface profiles by splines allows the detection of surface defects as regions of the surface that show a high distance relative to the approximating spline functions [32]. Specifically, the transform based on Hermite splines has a very convenient property, namely that the coefficients of a function corrupted with Gaussian noise follow a Gaussian distribution [63]. This property allows efficient denoising by simple thresholding of the coefficients. A cubic Hermite interpolator is a third order parametric polynomial curve $Z\left(t\right)=TM_{H}G_{H}$, where t∈[0,1], T is a row vector containing the coefficients of the third order polynomial function, $M_{H}$ is the square matrix form composed of the four Hermite basis functions, and $G_{H}$ is the column vector form composed of the interval endpoints values and their derivatives,

(13) $$\begin{array}{ccc} T & = & \left[\begin{array}{cccc} t^{3} & t^{2} & t & 1 \end{array}\right]\\ M_{H} & = & \left[\begin{array}{cccc} 2 & 1 & -2 & 1\\ -3 & -2 & 3 & -1\\ 0 & 1 & 0 & 0\\ 1 & 0 & 0 & 0 \end{array}\right]\\ G_{H} & = & {\left[\begin{array}{cccc} \rho (0) & \rho^{\prime }\left(0\right) & \rho (1) & \rho^{\prime }\left(1\right) \end{array}\right]}^{T} \end{array}$$

where ρ(0) and ρ(1) are the values at the boundaries of each interpolated curve, which may correspond to a surface fiber in our application, and $\rho^{\prime }\left(0\right)$ and $\rho^{\prime }\left(1\right)$ are their corresponding first derivatives with respect to *t*. A series of curve sampling intervals ${\left\{\rho_{k},\rho_{k}^{\prime }\right\}}_{k=1}^{n}$ can be interpolated by imposing boundary conditions such that the derivatives are continuous at the endpoints of neighboring intervals. These boundary conditions yield an interpolated curve consisting on cubic Hermite splines that is globally continuous and differentiable in (t(1),t(k)), i.e., it belongs to $C^{0}$ and $C^{1}$ [64].

However, in order to use the piece-wise cubic Hermite interpolation method, several considerations must be taken into account. On the one hand, the surface fiber recovered from the sensor must have a signal-to-noise ratio (SNR) high enough to allow data interpolation. On the other hand, according to the Nyquist–Shannon sampling theorem [65], the gradient data sampling frequency must be at least twice that of the highest frequency of the original signal. Thus, the accuracy of the surface gradient is determined by the distance between the sensor laser projection planes. The flatness defects produced by leveling processes are of a much lower frequency than the sensor sampling frequency. Therefore, in the absence of high frequency vibrations due to mechanical effects, the system has enough resolution to acquire the relevant flatness information.

## 5. Sources of Error

There are two different sources of error in the laser-camera triangulation process. One is the error in the estimation of the homography between the laser planes, and the other is the sub-pixel laser detection errors described in Section 3.1.

### 5.1. Measurement Errors Due to the Laser-Camera Triangulation

Due to the fact that the homography is estimated using several points, the errors due to this estimation may overcome the errors induced by the laser line intensity peak detection in the image. The segmentation errors of the laser points projected in the image plane have a normal distribution, zero mean, and a standard deviation σ. Therefore, the retroprojection errors propagate to the estimation of the real-world coordinates using the homography as follows:

(14) $${\tilde{p}}_{mm}=H\left({\tilde{p}}_{px}+{\tilde{\epsilon }}_{px}\right)$$

where H is the homography, ${\tilde{p}}_{mm}\in R^{2}$ are the homogeneous coordinates of the real triangulated point, ${\tilde{p}}_{px}\in R^{2}$ are the homogeneous coordinates of the segmented point in the image plane, and ${\tilde{\epsilon }}_{px}\in R^{2}$ is the reprojection error with the last row value equal to zero.

### 5.2. Measurement Errors Due to the Gradient Estimation

Gradient estimation errors are due to numerical differentiation, truncation, and round-off errors [66]. The error bounds between the truncated Taylor expansion of the 1D function derivative and the central difference computed at point $p_{mm}$ are:

(15) $$\left|E_{d}\right|\leq \left|\frac{h^{2}}{6}f^{\prime \prime \prime }\left(\xi \right)\right|+\left|\frac{\epsilon }{h}\right|$$

where $\left|\frac{h^{2}}{6}f^{\prime \prime \prime }\left(\xi \right)\right|$ is the Taylor expansion truncation error at point with *h* the step of the central difference in $p_{mm}$ and $\left|\frac{\epsilon }{h}\right|$ the round-off error.

Flatness calculation in rolled metal steel assumes that flatness defects can be approximated by sinusoidal waveforms, so that the maximum absolute derivative $\left|E_{s}\right|$ and the relative estimation errors η for a sinusoidal fiber along the unrolled metal sheet of amplitude *B* and period τ, respectively, are given by the following Equations:

(16) $$\begin{array}{ccc} \left|E_{s}\right| & \leq & \frac{B}{6}{\left(\frac{2\pi }{\tau }\right)}^{3}h^{2} \end{array}$$

(17) $$\begin{array}{ccc} \eta & \leq & \frac{1}{6}{\left(\frac{2\pi }{\tau }\right)}^{2}h^{2} \end{array}$$

## 6. Real-Time Surface Reconstruction Based on Hermite Interpolation

To inspect rolled products detecting flatness defects, it is necessary to scan the sheet as it moves along the processing line. The feeding rate of some lines reaches speeds up to 120m/min, posing stringent requirements for accurate real-time surface estimation.

**Figure 6:**
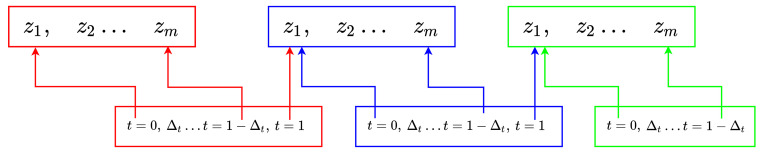
Scheme showing the signal partition scheme followed to allow Hermite splines’ interpolation.

To compute the noise-free reconstruction of one metal strip fiber $\hat{f}$ using piece-wise Hermite cubic interpolation, the fiber is uniformly partitioned into *n* intervals, each one discretized into *m* samples of its height measurement $z_{1},...,z_{m}$. Height derivatives $z_{1}^{\prime },...,z_{m}^{\prime }$ are computed using the information retrieved from the proposed two laser line sensor. Consecutive intervals must be continuous functions in $C_{0},C_{1}$. The boundary conditions of the signal intervals are set according to the computing strategy shown in Figure 6, i.e., the last measure of an interval is the first one for the next interval.

In Figure 6, Δt=1/m, and the value $z_{1}$ corresponding to the next interval is both calculated as the Hermite value for t=1 of the actual interval and also for t=0 of the next interval. The same computational scheme is applied to the derivatives, leading to the following linear equation $\hat{f}=G\left(f\right)A,$ which is constructed as follows:

(18) $$\begin{array}{cc} \hat{f}=\left[\begin{array}{c} \rho {}_{1}\\ \rho {}_{2}\\ \vdots \\ \rho {}_{n}\\ \rho_{1}^{\prime }\\ \rho_{2}^{\prime }\\ \vdots \\ \rho_{n}^{\prime } \end{array}\right] & {\left[\begin{array}{ccccc} A_{H} & 0 & 0 & \cdots & 0\\ 0 & A_{H} & 0 & \cdots & 0\\ \vdots & \vdots & \vdots & \vdots & \vdots \\ 0 & 0 & 0 & \cdots & A_{H}\\ A_{H}^{\prime } & 0 & 0 & \cdots & 0\\ 0 & A_{H}^{\prime } & 0 & \cdots & 0\\ \vdots & \vdots & \vdots & \vdots & \vdots \\ 0 & 0 & 0 & \cdots & A_{H}^{\prime } \end{array}\right]}_{\left(2n\times m)\times (4\times n\right)} \end{array}$$

where:

(19) $$\begin{array}{c} A_{H}={\left[\begin{array}{c} TM_{H}\left(t=0\right)\\ \vdots \\ TM_{H}\left(t=1-\Delta t\right) \end{array}\right]}_{m\times 4}\\ A_{H}^{\prime }={\left[\begin{array}{c} T^{\prime }M_{H}\left(t=0\right)\\ \vdots \\ T^{\prime }M_{H}\left(t=1-\Delta t\right) \end{array}\right]}_{m\times 4} \end{array}$$

and:

$$\begin{array}{cc} \rho_{i} & ={\left[z_{i1},\cdots ,z_{im}\right]}^{T}\\ \rho_{i}^{\prime } & ={\left[z_{i1}^{\prime },\cdots ,z_{im}^{\prime }\right]}^{T} \end{array}$$

$z_{ij}$ and $z_{ij}^{\prime }$ correspond to the measured height of the *j*th sample and its derivative at the *i*th interval of the fiber f, respectively. For a fiber $f_{i}$, these derivatives are computed as follows:

(20) $$\frac{\Delta f_{i}}{\Delta t}=\frac{\Delta f_{i}}{\Delta x}\frac{\Delta x}{\Delta t}$$

where Δx is the actual discretization step in the direction of the surface translation. Finally, $G\left(f\right)$ contains the end points and its derivatives for each sampled interval along fiber f, which can be calculated by linear methods. Real-time computation using the previous scheme can be extended to all the fibers captured over the total metal sheet surface, resulting in:

(21) $$\left[{\hat{f}}_{1}\cdots \hat{f}{}_{K}\right]=A\left[G\left(f_{1}\right)\cdots G\left(f{}_{K}\right)\right]$$

where:

(22) $$G\left(f_{k}\right)={\left[\rho {}_{i1}^{T},\ldots ,\rho {}_{in}^{T}|\rho {}_{i1}^{{}^{\prime }T},\ldots ,\rho {}_{in}^{{}^{\prime }T}\right]}^{T}$$

The matrix A is common to all longitudinal metal sheet fibers ${\left\{f_{k}\right\}}_{k=1}^{K}$; therefore, it is computed only once, facilitating real-time implementation.

## 7. Results

We apply the proposed Hermite polynomial filtering and reconstruction method to both simulated and real data in order to test its ability to remove high frequency noise due to mechanical manipulation of the metal sheet.

### 7.1. Synthetic Data Results

**Figure 7:**
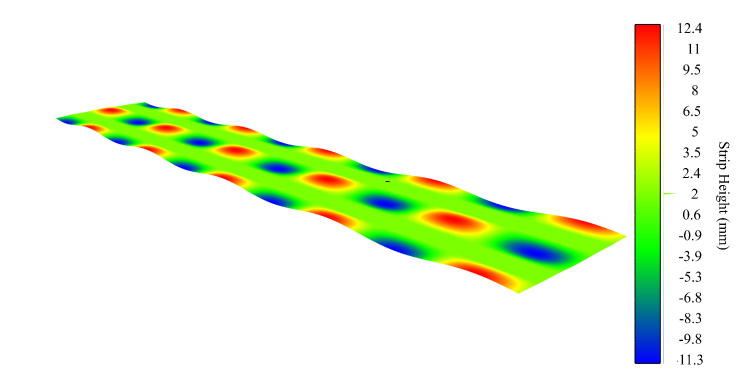
A noise-free synthetic surface showing center buckles and wavy edge defects.

We created a synthetic surface showing two of the most common defects in a roll leveler processing line, namely center buckles and wavy edges. The period and amplitude of these sinusoidal flatness defects are 1800mm and 12mm on the translation axis of the metal sheet and 1500mm and 12mm across the width of the metal sheet, respectively. The simulated surface is shown in Figure 7.

The first computational experiment is carried out over the synthetic surface described above without additional noise, in order to simulate an ideal production line and check the accuracy of our method. The surface filtering has been made with a sampling interval for the Hermite interpolation of $\Delta_{x}=3\;$mm. Each fiber interval contains 33 samples, which corresponds to the distance between lasers ($\Delta_{d}$). The total length of the sheet is 9000mm. The proposed Hermite filtering method maximum surface reconstruction error is 0.64μ m with standard deviation σ=0.16μm. The relative error in the gradient computation according to the theoretical maximum error defined by Equation(16) is 0.507% for $h=\Delta_{d}/2$.

In the second computational experiment, we introduced different kinds of noise to simulate the vibrations induced in the sheet metal as a result of the mechanical elements of the roll leveler line. Additionally, we introduced another noise source controlled by a single parameter that simulates the triangulation error of the 3D sensor. The noise sources that characterize the mechanical vibrations have an amplitude of 2.5mm following a Gaussian distribution. A high frequency sinusoidal signal with a maximum amplitude of 0.5mm and a spatial period of 50mm is also added in order to simulate the eccentricity of the rolls over which the sheet is displaced. The noise introduced as a consequence of the laser peak detection error has a standard deviation of 1/16 pixel, which corresponds to typical camera laser peak detection errors. The results obtained comparing the reconstructed surface to the theoretical noise-free surface are a maximum estimation error of 0.35mm and a standard deviation of 0.12mm. These errors meet the standards currently used to measure flatness quality in roll leveled products.

**Figure 8:**
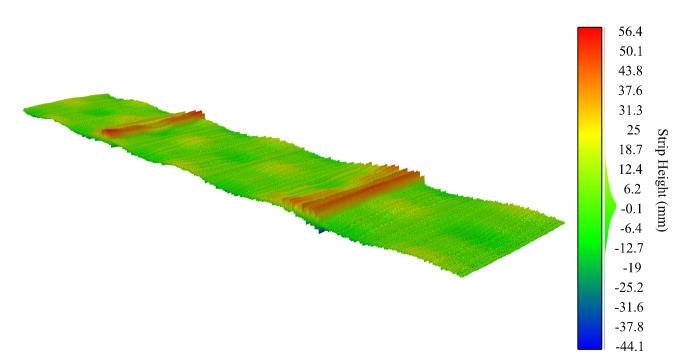
The synthetic surface after adding the effect of vibrations induced by different mechanical sources such as the shearing station (noise sources added).

In the third experiment, we added a localized damped sine wave along the longitudinal axis of the synthetic sheet with an initial amplitude of the envelope of 50mm, a decay constant of 0.07, and a period of 10mm. This signal models the vibrations induced by a sheet metal cutting station located near the scanning area, as shown in Figure 8. The existing literature rarely considers the effects produced by processes subsequent to the roll leveling. Using our filtering method, we obtain a maximum error of 1.15mm and a standard deviation of 0.38mm.

**Figure 9:**
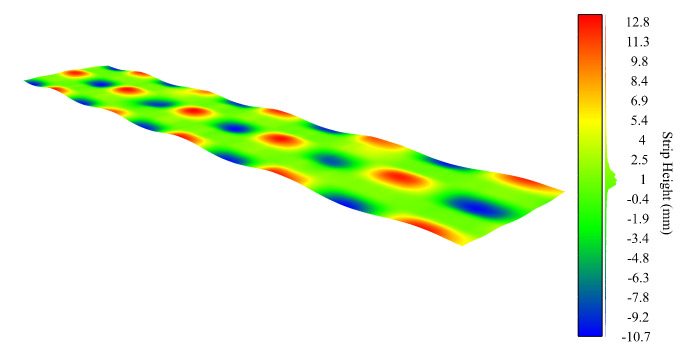
3D representation of the theoretical surface results after applying the proposed filtering method.

**Figure 10:**
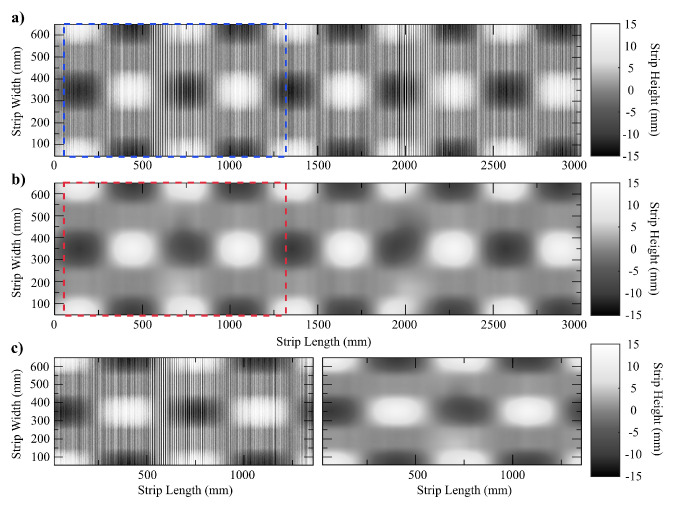
Visualization as a grayscale image of the synthetic surface results of the interpolation with Hermite splines. (**a**) Synthetic surface corrupted with high frequency noisy vibrations; (**b**) filtered surface; (**c**) a close up view of a region in both images. Intensity corresponds to height relative to the mean of the surface. White = positive; dark = negative.

These results show that the Hermite interpolation filtering method is very robust and that it enables an accurate and reliable surface estimation despite the presence of external noise sources, improving over other methods based on FIR techniques, e.g., Butterworth, Savitzky–Golay, etc. Figure 9 shows the the estimated filtered surface from the noisy surface data of Figure 8. Maximum errors occur at the high amplitude damped noise regions, due to the loss of reliable surface height information obtained by the sensor. These results are also shown in Figure 10, where the raw data retrieved from the sensor are shown first, the estimated surface using our method is presented in the middle, and finally, two insets provide more detailed information from the dashed areas colored in red and blue color, respectively.

These results show that the flatness sensor and the proposed filtering method are both robust and accurate enough against different noises sources, in particular to localized high amplitude noises produced by a simulated cutting station placed after the rolling leveler and the devised 3D sensor.

For a quantitative evaluation of the improvement achieved by our proposal, we implemented the following approaches for surface denoising:

1. A moving-average filter featuring a sliding window of 33 samples in length.
2. A third order low-pass Chebyshev Type II filter [67] with 33 dB of stopband attenuation and a stopband edge frequency of 0.02 specified in normalized frequency units.
3. A third order Savitzky–Golay FIR smoothing filter [48] with a frame length of 99 samples.
4. A third order Butterworth IIR digital filter [68] with a cutoff frequency for the point 6 dB below the passband value of 0.01 specified in normalized frequency units.

Table 1 presents the comparative results of the implemented denoising methods over the synthetic surface discussed above. Improvements in MAE achieved by our approach range from 45% relative to the Butterworth filter approach up to 50% relative to the Savitzky–Golay approach. Regarding the RMSE, our approach improves by 41% over the second best approach, and up to 49% over the Chebyshev filter.

### 7.2. Results on Real Industrial Data

**Figure 11:**
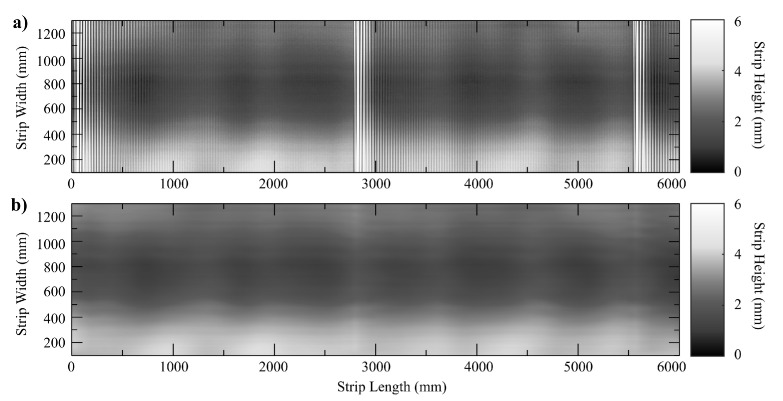
(**a**) Sensor raw data for a S235JR steel coil with observed high frequency transient noisy waves and background noise. (**b**) Denoised sensor data using the proposed Hermite interpolation filtering method.

**Figure 12:**
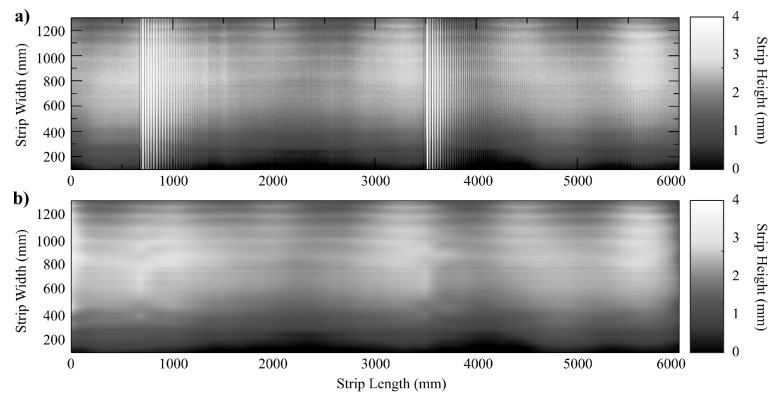
(**a**) Sensor raw data for a S500MC high yield steel coil with with observed periodic transient impulses and background noise. (**b**) Denoising results of the sensor raw data using the proposed Hermite interpolation filtering method.

During the experimental data acquisition, we used sheet samples from two kinds of steel S235JR steel coils (thicknesses of 3mm, Young’s modulus E=205GPa, Poisson’s ratio ν=0.301, yield stress $\sigma_{0}=215\;$MPa) and S500MC high yield steel coils (a thickness of 8mm, Young’s modulus E=210GPa, Poisson’s ratio ν=0.304, yield stress $\sigma_{0}=500\;$MPa), which are middle carbon steels manufactured by rolling. They are annealed and skin passed. The coil specimens have a length of about 800 m. The sensor takes measurements along 3000 mm in each measurement cycle. The widths are in the range $\left[800,2300\right]$ mm. The behavior of these specimens was characterized by uniaxial tensile tests conducted at room temperature. The raw depth information from the sensor for samples of these two different kinds of steel coil are visualized as grayscale images in Figure 11a and Figure 12a. Flatness information is highly corrupted by high frequency noisy vibrations from the cutting station and other mechanical effects on the steel sheet. Namely, the cutting station produces high amplitude/high frequency noisy waves on the metal strip every *T* seconds depending on the cutting length program, and the conveyor belt produces overall high frequency waves. The resulting interference pattern is a rather complex spatial form of noise, hindering the detection of flatness defects.

**Figure 13:**
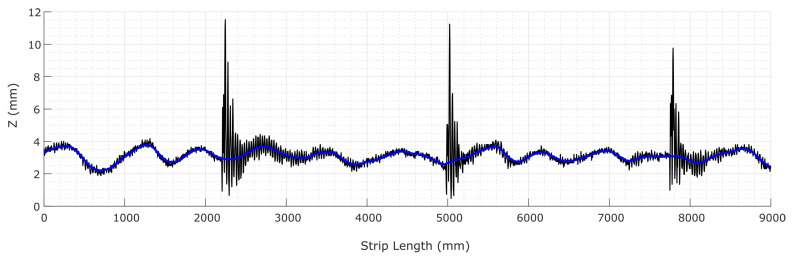
An instance of longitudinal fiber reconstruction (light blue line) by Hermite interpolation from the raw data of Figure 11 (dark line).

The proposed flatness measuring sensor succeeds at acquiring the surface information at the real-time production speed of 30m/min. The Hermite polynomial filtering procedure manages to successfully retrieve the surface features of the material and the denoised flatness measures, as shown in Figure 11b and Figure 12b, depicting the smooth reconstructed surface after the noisy waves have been removed. In Figure 12b, filtering unveils center buckles, i.e., another kind of common flatness defect in which sinusoidal waves restrain the central fibers of the metal sheet. Finally, Figure 13 shows the reconstruction of one of the surface fibers from the raw data of Figure 11.

### 7.3. Limitations of the Real Data Results

Estimation of the measurement error on real sensor measures remains very challenging. Actually, there is no way to carry out a fair comparison between the measures obtained by our sensor with those of a precision measuring instrument, such as a coordinate-measuring machine (CMM). Measuring a test specimen of a metal sheet out of the roll leveling machine with a CMM has some inherent bias, because the sheet is released from tensile and traction stresses, while our optical sensor measures the strip under stress. Therefore, the flatness measures yielded by a CMM cannot be fairly compared with those of an online sensor measuring the actual production.

The measuring device and the filtering method proposed in the manuscript solves flatness data reconstruction in a real-time framework. In fact, it takes an average of 0.35 s seconds to filter a metal sheet of 6000mm in length by 2300mm in width. The sampling distance in the longitudinal direction (rolling direction) is 3mm and in the transversal direction is 2mm; thus 1150 fibers are computed concurrently in this time. The time that this sheet will take to go under the measuring sensor at maximum speed of 120m/min is about 3s, so that real-time measurement is feasible in operational conditions. Furthermore, our system uses a first in first out (FIFO) queue to store acquired data until the filtering process finishes.

The proposed sensor relies on the motion of the steel sheet for the measurement. Doing the measurements off-line for comparison with contact sensor measurements carried out off-line would require building up a complete setup where the sensor is displaced over the cut steel plate. We carried out some comparisons of the measurements done by the sensor oonline with off-line measurements by shape measurement rolls. The differences were on the order of 0.25 mm, within standard industry resolution requirements. It must be taken into account that the off-line contact sensor measurements do not suffer from the transport tension, cutting, and other vibration sources.

Finally, this method can be used to concatenate as many processed sheets as needed. In order to ensure the $C_{0},C_{1}$ continuities between consecutive processed sheets, the Hermite surface reconstruction method can be easily enhanced, by imposing as boundary conditions for the actual piece the height values and the derivatives of the previous piece.

## 8. Conclusions

In this paper, we propose a hardware-software system that carries out in real time the estimation of the flatness of a metal strip moving below the unrolling mill that flattens a roll of steel cutting it into sheets of predefined lengths. Oonline quality inspection of the unrolled metal strip before cutting is achieved by this system. The standard flatness anomalies to be detected are wavy edges, center buckles, and bow defects, which are low frequency variations in the height of the metal surface. The mechanical manipulation of the metal, such as the jerking due to the strip pulling and tugging, and the cutting process, induces high frequency variations in surface height that are successfully removed by our surface reconstruction software system, which is based on a Hermite interpolation approach.

The nonlinear combination of the different noise sources on this particular process means that filtering the sensor signals with the desired precision cannot be achieved by conventional linear filtering techniques. We validated the filtering approach on synthetic and real industrial operation data, showing significant improvement over linear conventional approaches. In the comparison with Savitzky–Golay, Chebyshev, and Butterworth filters applied to the reconstruction of the surface after noise addition, we achieved more than a 41% improvement. Furthermore, we were able to assess the accuracy of the sensor against off-line contact sensor measurements, reporting an average error of 0.25 mm within standard industry accuracy requirements.

The proposed sensor and surface reconstruction system can pave the way for online closed-loop control systems, low cost real-time flatness quality inspection, and high efficiency and quality manufacture of rolled steel products. More extensive and detailed analysis of the impact of gradient estimation numerical errors should be carried out.

## Figures and Tables

**Table 1:** Comparative results of our approach with other conventional denoising approaches. MAE = mean absolute error; MaxAE = maximum absolute error; STD = standard deviation of the absolute error; RMSE = root mean squared error.

Method	MAE	MaxAE	STD	RMSE
Hermite (ours)	0.413	1.15	0.38	0.459
Butterworth	0.760	4.423	0.735	0.781
Savitzky–Golay	0.842	6.436	0.779	0.853
Moving Average	0.801	5.463	0.928	0.865
Chebyshev Type II	0.828	5.040	0.828	0.903

## Abbreviations

The following abbreviations are used in this manuscript:

ASTM	American Society for Testing and Materials
